# Renal Affections: Their Diagnosis and Pathology

**Published:** 1851-01

**Authors:** 


ART. VI.—*Renal Affections:* their diagnosis and pathology. By CHAS. FRICK, M. D.

We have taken every opportunity in our power, to urge upon medical men the necessity of educating themselves in the use of the microscope. A number of valuable Medical works are now before the profession, which are almost unintelligible, in some important particulars, to those ignorant of microscopic investigations. And the details of microscopic medical knowledge are continually increasing in interest, and will be greatly extended. Stanley, of St. Bartholomews, and Bransby Cooper have contributed a great deal of invaluable surgical information, by the aid of the microscope; Hassall has rendered valuable service to medical science, by his "microscopic anatomy of the human body, in health and disease," and all departments of medical research are fully awakened to the importance of the use of the microscope. But we have not space now to dwell upon the subject.

The work before us is one well worthy the attention of the profession. A medical man may, with this work, and Bowman's Chemistry, and a good microscope, all properly used, soon find himself rising in the scale of medical knowledge. He may soon reach a point of elevation that may give him full and excellent views of the rich landscape that spreads out before him, of whose existence he would scarcely have known anything except for the agency of this admirable instrument.

Dr. Frick's admirable work treats of the following subjects: "Precautions to be observed in obtaining a specimen of urine, with instructions for a superficial examination at the bedside." "Directions for a more exact examination of the different ingredients of the urinary secretion." "On the microscopic examination of the urine." "On the pathology of the different substances found in the urinary secretion." "On the pathology and therapeutical indications of the more important elements of the urinary secretions." "On the analysis of calculi."

Dr. Frick handles these various subjects very admirably, and has compressed a large amount of practical knowledge within the compass of 179 pages. We cordially thank Dr. Frick for this contribution to the progress of Practical Medicine, and we regret that our very limited space in the Bibliographical department of this number of the *Journal* forbids a more extended notice.

